# Editorial: Rickets and osteomalacia, from genes to nutrition

**DOI:** 10.3389/fendo.2023.1141888

**Published:** 2023-01-19

**Authors:** Artemis Doulgeraki, Michaël R. Laurent

**Affiliations:** ^1^ Department of Bone and Mineral Metabolism, Institute of Child Health, Agia Sophia Children’s Hospital, Athens, Greece; ^2^ Centre for Metabolic Bone Diseases, University Hospitals Leuven, Leuven, Belgium; ^3^ Geriatrics Department, Imelda Hospital, Bonheiden, Belgium

**Keywords:** genetics, hypophosphatemia, nutrition, osteomalacia, rickets

Rickets in children and osteomalacia in adults are metabolic bone disorders which can be genetic or acquired [most often nutritional or drug-related (1)]. During the last decade, we have seen considerable advances on the diagnosis and treatment of genetic rickets, given the enormous progress in genetics, which in turn allows targeted medications (2). On the other hand, nutritional rickets remains a pressing public health issue, especially in view of the recent refugee crisis in many countries, *i.e.* the increase of mobile populations (3). Some recent large population-based studies estimate a prevalence of rickets of up to 10% in children, even in sunny climates (4). Of note, nutritional rickets is entirely preventable and treatable.

The vivid research interest in all forms of rickets prompted the production of this special issue, whose main scope was to highlight important aspects of these disorders and give guidance on everyday practice, as there are still many unanswered questions.

Nutritional rickets has not only medical but also social and political aspects. This special issue contains two articles with translational research on this topic and hopefully a positive impact on everyday practice. The first one, by Uday and Högler describes the biochemical profile of household members of index cases with nutritional rickets. The concept of “biochemical osteomalacia” is not new (5) but is gaining ground also in international literature (6), since the gold standard of bone biopsy remains invasive and expertise in bone histomorphometry remains lacking in many countries (1). Not surprisingly, Uday and Högler found that the majority of mothers and siblings of rickets index cases also had considerable vitamin D deficiency and biochemical rickets/osteomalacia. The author’s “take home” message is to provide vitamin D supplementation to the whole family for as long as the risk factors remain. Indeed, targeting of high risk family members for further testing and treatment, *i.e.* mothers of infants, family members on calciopenic diets and symptomatic individuals, appears sensible and is likely cost-effective. Detailed history taking for symptoms and risk factors is important, because it enables identification of the high-risk groups for rickets.

The second article, by Högler et al. also has significant public health implications. In neonatal dried blood spots from a large study in the Midlands (U.K.), 35.7%had vitamin D deficiency (defined as 25-hydroxyvitamin D < 30 nmol/L, *i.e.*<12 µg/l) and 33.7% had vitamin D insufficiency (defined as 25-hydroxyvitamin D 30-50 nmol/L *i.e.*12-20 µg/l), leaving only 30.6% with vitamin D sufficiency. The use of newborn screening cards for this purpose highlights an important means of research in this vulnerable population and offers new insights on research methodology in the field of vitamin D disorders. A comprehensive analysis of seven different social domains in relation to vitamin D levels is described in an effort to present the social and political aspects of nutritional rickets and is certainly thought provoking. Special emphasis is given on the living conditions, therefore appropriate questions should be asked during history taking (indoors: *e.g.* quality of housing, outdoors: *e.g.* air quality, activities undertaken by the pregnant mothers). The paper also emphasizes the need for more efficient care of pregnant women with appropriate vitamin D supplementation, universal food fortification with vitamin D and initiatives to overcome social disparities.

With regards to genetic rickets, our Research Topic includes two articles focusing on hypophosphatemic conditions. Xu et al. describe the clinical and genetic characteristics of 29 Chinese patients with X-linked hypophosphatemia (the commonest genetic cause of rickets) (7). The clinical characteristics are very similar to reports from Western or other Chinese cohorts, confirming the universal clinical disease presentation (8). Several new underlying aberrations in the *PHEX* gene are described, which expand the list of confirmed pathogenic mutations. Orthopedic surgeries in this cohort are described with variable success, underscoring the need to better understand the optimal timing and approaches for surgery in this condition (2) (8), Larger international registry studies could fill this critical gap.

Choe et al. provide a case report and literature review of autosomal recessive hypophosphatemic rickets type 2, caused by mutations in the ectonucleotide pyrophosphatase/phosphodiesterase 1 (*ENPP1*) gene. Although biallelic mutations in this gene may cause generalized arterial calcification of infancy, early-onset hearing loss, ossification of posterior longitudinal ligament and pseudoxanthoma elasticum (2), the child in this case presented with hypophosphatemic rickets alone. Despite good compliance with conventional therapy, orthopedic surgery was required. This rare case illustrates the unmet medical need in this condition, for which therapies targeting pyrophosphate metabolism are under development (9). Of note, other reports underline that heterozygous mutations in this gene may present with diffuse idiopathic hyperostosis (7). Thus, the burden of *ENPP1* mutations may be much larger than previously appreciated.

Finally, the review article by Ogunmwonyi et al. summarizes our current understanding of the genetic and epigenetic contributions to the development of nutritional rickets. Indeed, gene-environment interactions have long been hypothesized to explain why within certain at risk populations (*e.g.*with low-calcium diets or veiling for example), only a subgroup of children develops rickets clinically. For example, twin studies summarized in this review suggest that 25-hydroxyvitamin D concentrations may be genetically determined by about 50% (range 23-80%) (14). Single-nucleotide polymorphisms in several vitamin D metabolism-related genes have also been implicated in the development of nutritional rickets (14). More worryingly is our emerging understanding how early-life exposure to vitamin D deficiency may influence epigenetic signals, determining growth and growth plate signaling later in life. A prime example is provided by the recent Maternal Vitamin D Osteoporosis Study (MAVIDOS) randomized trial (10). In this randomized trial, maternal vitamin D supplementation during pregnancy suppressed CpG methylation (a marker for epigenetic silencing) at the locus for retinoid-X-receptor-alpha (which dimerizes with the vitamin D receptor to regulate gene expression) in offspring umbilical cord samples (10). Long-term follow-up of this cohort has already shown beneficial effects of vitamin D supplementation during pregnancy on offspring’s bone health (11). Further research in this area is eagerly awaited.

To summarize, we can conclude that rickets and osteomalacia are not “forgotten” diseases belonging in history textbooks. Both genetic and nutritional forms remain highly relevant today and in the near future. For the genetic forms, several promising therapeutics have emerged in recent years, and more are under active development, holding promise for patients with rare bone diseases. For the acquired forms, definitions of “biochemical osteomalacia”, if prospectively validated against bone biopsies, could facilitate clinical and epidemiological research. Public health strategies are however required to eradicate nutritional rickets, an entirely preventable condition with a high disease burden, on a global scale.

## Author contributions

Both authors were responsible for the conception, writing of the manuscript, revision of content and approve the submitted version.
